# 1460. Respiratory Syncytial Virus (RSV)-Related Clinical Events among a Medicare-Insured Population in the United States

**DOI:** 10.1093/ofid/ofac492.1287

**Published:** 2022-12-15

**Authors:** Jessica K K DeMartino, Marie-Hélène Lafeuille, Bruno Emond, Carmine Rossi, Jingru Wang, Stephanie Liu, Patrick Lefebvre, Girishanthy Krishnarajah

**Affiliations:** Janssen Scientific Affairs, LLC, Titusville, New Jersey; Analysis Group, Inc., Montreal, Quebec, Canada; Analysis Group, Inc., Montreal, Quebec, Canada; Analysis Group, Inc., Montreal, Quebec, Canada; Analysis Group, Inc., Montreal, Quebec, Canada; Analysis Group, Inc., Montreal, Quebec, Canada; Analysis Group, Inc., Montreal, Quebec, Canada; Janssen Scientific Affairs, LLC, Titusville, New Jersey

## Abstract

**Background:**

RSV is a contagious pathogen that is often underrecognized in older adults. While the general burden of RSV has previously been assessed, little is known on the frequency and factors associated with RSV-related clinical events in older adults.

**Methods:**

Patients ≥60 years old with a diagnosis code for RSV (index date) and ≥6 months of enrollment pre-index (baseline) were analyzed using claims data from the 100% Medicare database (2007-2019). The following RSV-related clinical events were assessed during the up-to-6-month post-index period: acute respiratory failure, chronic respiratory disease (asthma or chronic obstructive pulmonary disease), congestive heart failure, dyspnea, hypoxia, non-RSV lower/upper respiratory tract infection, and pneumonia. Patients were at risk of developing each clinical event if they did not already have the event at baseline. A stepwise Poisson regression model was used to identify baseline predictors of having ≥1 clinical event.

**Results:**

A total of 175,392 patients were included (mean age: 79.0 years, 64.8% female, 78.4% white, 76.9% had ≥1 RSV-related clinical event at baseline). During the up-to-6-month period following RSV infection, 47.9% had ≥1 incident RSV-related clinical event (**Figure**). The mean (median) time to a clinical event was 1.0 (0.1) month. Having an event was more likely for patients with baseline conditions (coronary artery disease, diabetes, or one of the above RSV-related clinical events [except pneumonia and asthma]) or with chemotherapy, chest x-ray, organ transplant, anti-asthmatic use, or bronchodilator use at baseline (incidence rate ratio [IRR] range=1.06 [bronchodilators] to 1.80 [chest x-ray], all *P*< .05); having an event was less likely for patients with one of the following: lower age, female gender, baseline influenza or RSV test, baseline use of antibiotics, or baseline use of influenza agents (IRR range=0.63 [antibiotics] to 0.92 [baseline RSV test], all *P*< .05).

**Figure:**
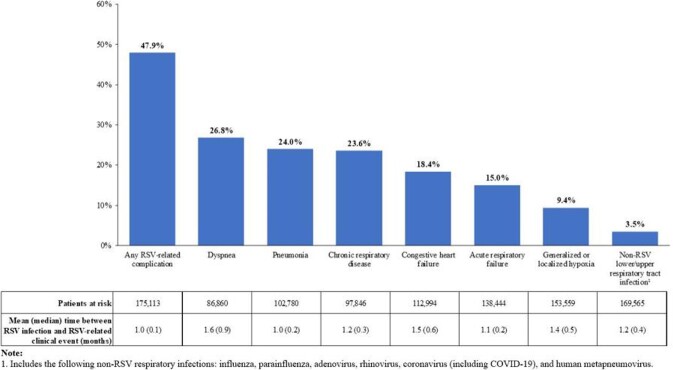
RSV-related clinical events among Medicare beneficiaries ≥60 years old

**Conclusion:**

Almost half of patients ≥60 years old had an RSV-related clinical event within 1 month of RSV infection; patients with pre-existing conditions (≥75% of patients) were at higher risk of an event. These findings highlight that many older adults with RSV experience significant clinical events that burden both the patient and the healthcare system.

**Disclosures:**

**Jessica K. K. DeMartino, PhD**, Janssen Scientific Affairs, LLC: Employee of Janssen Scientific Affairs, LLC **Marie-Hélène Lafeuille, MSc**, Janssen Scientific Affairs, LLC: Advisor/Consultant **Bruno Emond, MSc**, Janssen Scientific Affairs, LLC: Advisor/Consultant **Carmine Rossi, PhD**, Janssen Scientific Affairs, LLC: Advisor/Consultant **Jingru Wang, BA**, Janssen Scientific Affairs, LLC: Advisor/Consultant **Stephanie Liu, MS**, Janssen Scientific Affairs, LLC: Advisor/Consultant **Patrick Lefebvre, MSc**, Janssen Scientific Affairs, LLC: Advisor/Consultant **Girishanthy Krishnarajah, MBA**, Janssen Scientific Affairs, LLC: Employee of Janssen Scientific Affairs, LLC.

